# Association Between Financial Incentives in Medicare's Hospital Readmissions Reduction Program and Hospital Readmission Performance

**DOI:** 10.1001/jamanetworkopen.2020.2044

**Published:** 2020-04-03

**Authors:** Geoffrey J. Hoffman, Olga Yakusheva

**Affiliations:** 1 Department of Systems, Populations and Leadership, University of Michigan School of Nursing, Ann Arbor; 2 Institute for Healthcare Policy and Innovation, University of Michigan, Ann Arbor

## Abstract

**Importance:**

The strongest evidence for the effectiveness of Medicare's Hospital Readmissions Reduction Program (HRRP) involves greater reductions in readmissions for hospitals receiving penalties compared with those not receiving penalties. However, the HRRP penalty is an imperfect measure of hospitals' marginal incentive to avoid a readmission for HRRP-targeted diagnoses.

**Objectives:**

To assess the association between hospitals’ condition-specific incentives and readmission performance and to examine the responsiveness of hospitals to condition-specific incentives compared with aggregate penalty amounts.

**Design, Setting, and Participants:**

This retrospective cohort analysis used Medicare readmissions data from 2823 US short-term acute care hospitals participating in HRRP to compare 3-year (fiscal years 2016-2019) follow-up readmission performance according to tertiles of hospitals' baseline (2016) marginal incentives for each of 5 HRRP-targeted conditions (acute myocardial infarction, heart failure, chronic obstructive pulmonary disease, pneumonia, and hip and/or knee surgery).

**Main Outcomes and Measures:**

Linear regression models were used to estimate mean change in follow-up readmission performance, measured using the excess readmissions ratio, with baseline condition-specific incentives and aggregate penalty amounts.

**Results:**

Of 2823 hospitals that participated in the HRRP from baseline to follow-up, 2280 (81%) had more than 1 excess readmission for 1 or more applicable condition and 543 (19%) did not have any excess readmissions. The mean (SD) financial incentive to reduce readmissions for incentivized hospitals ranged from $8762 ($3699) to $58 158 ($26 198) per 1 avoided readmission. Hospitals with greater incentives for readmission avoidance had greater decreases in readmissions compared with hospitals with smaller incentives (45% greater for pneumonia, 172% greater for acute myocardial infarction, 40% greater for hip and/or knee surgery, 32% greater for chronic obstructive pulmonary disease, and 13% greater for heart failure), whereas hospitals with no incentives had increases in excess readmissions of 4% to 7% (median, 4% [percentage change for nonincentivized hospitals was 3.7% for pneumonia, 4.2% for acute myocardial infarction, 7.1% for hip and/or knee surgery, 3.7% for chronic obstructive pulmonary disease, and 3.7% for heart failure]; *P* < .001). During the study period, each additional $5000 in the incentive amount was associated with a 0.6– to 1.3–percentage point decrease, or up to a 26% decrease, in excess readmissions (*P* < .001). Regression to the mean explained approximately one-third of the results depending on the condition examined.

**Conclusions and Relevance:**

The findings suggest that improvements in readmission avoidance are more strongly associated with incentives from the HRRP than with aggregate penalty amounts, suggesting that the program has elicited sizeable changes. Worsened performance among hospitals with small or no incentives may indicate the need for reconsideration of the program's lack of financial rewards for high-performing hospitals.

## Key Points

**Question:**

Are financial incentives from Medicare’s Hospital Readmissions Reduction Program associated with hospital readmission performance?

**Findings:**

This cohort study using Medicare performance data from 2823 hospitals from 2016 to 2019 found that hospitals with greater incentives for readmission avoidance had larger decreases in excess readmission, whereas hospitals with no incentives had increases in excess readmissions across Hospital Readmissions Reduction Program–targeted conditions.

**Meaning:**

The findings suggest that the penalty incentives in the readmissions program were associated with improvements in readmission avoidance.

## Teaser

This cohort study assesses the association of incentives from Medicare’s Hospital Readmissions Reduction Program with hospital readmission performance from 2016 to 2019.

## Introduction

Medicare's Hospital Readmissions Reduction Program (HRRP) has received increasing criticism for the size and distribution of its penalties, which have a mean of $200 000 per hospital, or $500 million overall annually.^1,2,3^ These penalties have been disproportionate, with larger penalties for hospitals serving disadvantaged patients, raising concerns about underresourced hospitals’ opportunities to invest in prevention.^4,5,6^ The larger the penalty, particularly for hospitals with fewer resources, the less ability hospitals might have to address quality issues,^7,8^ resulting in widening performance gaps between well-resourced hospitals and those with fewer resources. There have also been concerns about whether hospitals have responded to the HRRP or whether instead observed decreases in readmissions reflect nonperformance factors.^9^

However, the penalty may not be the most important factor associated with hospital responsiveness to HRRP. Because excess readmissions in a given year affect HRRP penalties in future years, hospital revenue for each patient consists of reimbursement for a current diagnosis plus the marginal effect on the future HRRP penalty.^10^ This means that incentives for HRRP reflect the marginal patient revenue for a readmission and penalty dollars that can be avoided on the margin.^10,11^ Given differences in hospitals’ marginal patient revenue, current penalty amounts, and future penalty exposure, there is considerable heterogeneity in incentives, ranging from approximately $10 000 to $60 000 for each avoided readmission.^12^ Incentives at underresourced hospitals are often smaller because, for each avoided readmission, they forego more Medicare supplemental payments for caring for vulnerable patients and employing medical residents.^11^

Previous research has reported an association of improvements in readmission rates with HRRP,^13^ including narrowing gaps between heavily penalized and underresourced hospitals compared with other hospitals.^5,14,15^ Desai et al^16^ found 0.5– to 1–percentage point greater reductions for penalized hospitals compared with nonpenalized hospitals, suggesting that HRRP was associated with improvements. However, unlike aggregate penalties, the incentives of HRRP are specific to targeted diagnoses and vary according to hospitals’ reimbursement levels and factors beyond being penalized or not; for instance, both highly penalized hospitals above the penalty cap and lightly penalized hospitals near the penalty floor have more limited incentives than other hospitals. We assessed the association between hospitals’ condition-specific marginal incentives and their readmission performance. With this approach, we aimed to inform policy makers about the extent to which the program elicited expected responses.

## Methods

### Data

For this cohort study, we used a data set containing incremental financial incentives for readmission avoidance and hospital performance from fiscal year (FY) 2016 through FY 2019. The study underwent review and approval from the University of Michigan, Ann Arbor, institutional review board. The data used were publicly available, and informed consent from hospitals was not required. This study followed the Strengthening the Reporting of Observational Studies in Epidemiology (STROBE) reporting guideline.

Yakusheva and Hoffman^12^ previously derived FY 2016 financial incentives information using readmissions and hospital information from FY 2011 to FY 2014 Medicare data files.^17,18^ We merged FY 2017, FY 2018, and FY 2019 Readmissions Supplemental Data Files with the FY 2016 incentives for 2465 hospitals with excess readmissions for any of 5 targeted conditions: acute myocardial infarction (AMI), heart failure, chronic obstructive pulmonary disease (COPD), pneumonia, and total hip and/or knee surgery. For hospitals without any incentives in FY 2016, incentives were set to 0. Therefore, the combined data set had hospitals’ baseline (2016) incentive amounts and follow-up (2017-2019) HRRP performance. We excluded any hospitals within each targeted condition that did not report readmissions data (excess readmission ratio [ERR], defined as a hospital’s condition-specific ratio of predicted to expected readmissions) for the final year of follow-up (2019).

Yakusheva and Hoffman^12^ previously computed marginal incentives as a function of hospitals’ reimbursement for a current diagnosis plus their marginal effect on the future HRRP penalty.^10^ Reimbursement for a current diagnosis consisted of the difference between the Medicare reimbursement that the hospital forfeited minus the savings from resources not used to care for the readmitted patient. For instance, for AMI, hospitals could save approximately $19 000 in future penalties but would forfeit approximately $2000 in marginal patient revenue (readmission reimbursement minus patient care costs), resulting in net reimbursement of approximately $17 000 for an avoided readmission. Heterogeneity in these amounts (the interquartile range for AMI was $12 826-$22 219) reflected differences in hospitals’ base operating payments adjusted for the wage index and policy payments (eg, wage index and disproportionate share hospital payments). The marginal effect on the future HRRP penalty was derived using a calculus-based derivation of the HRRP formula as a function of condition-specific volumes of index discharges, operating payments, and numbers of actual, predicted, and expected readmissions. Heterogeneity in these effects indicated that an avoided readmission would not reduce their penalties or otherwise financially benefit some hospitals, given the program's penalty cap (3% maximum) and floor (no reward for above-average performance), because hospitals still above the cap after an avoided readmission obtain 0 penalty savings whereas hospitals near the floor receive only partial credit for an avoided readmission. The eAppendix in the Supplement gives additional details; the mathematical derivation of the incentive is available elsewhere.^12^

### Other Measures

With HRRP, readmission performance is assessed using the ERR.^12^ Hospitals with more predicted than expected readmissions (ERR >1) for any applicable condition will incur an HRRP penalty applied to each Medicare discharge during the fiscal year (eg, hospitals with an FY 2016 ERR >1 would be penalized for each discharge that year). Hospitals with better-than-expected performance (ERR ≤1) are not penalized but also do not receive any financial reward.

### Statistical Analysis

We first evaluated the heterogeneity in hospital incentives by applicable condition with histograms. Then, we plotted the incentives by the total HRRP penalty amount. Next, we divided hospitals with a baseline ERR more than 1 into tertiles by incentive amount for each of the 5 applicable conditions. For each condition separately, hospitals in the first tertile had the smallest financial incentive and hospitals in the third tertile had the largest incentive to avoid a readmission. A fourth group included those hospitals (baseline ERR ≤1) with no incentives in 2016.

Using ERRs, we then examined the change in hospital performance over time from baseline (2016) to follow-up (2019) overall and according to baseline incentive tertile. Because ERRs for a given year are estimated by the Centers for Medicare & Medicaid Services using 3 years of prior performance data (eg, FY 2016 ERRs are computed using FY 2011-2014 data), these assessments measure whether hospitals who had early poorer performance that could generate penalties would respond by improving performance in later years. Paired *t* tests were used to assess the overall changes in ERRs within condition. We expected that, within each condition, hospitals with the largest incentives (in the third tertile) would have the greatest reduction in readmissions. Analysis of variance tests were used to assess whether, within conditions, there were differential changes in performance across incentive tertiles as well between tertiles of hospitals with incentives and hospitals with no incentives.

We also examined responsiveness to the incentives vs the aggregate penalty. To do so, we estimated ordinary least squares regression models to estimate the change in ERR according to $5000 increases in the incentive (which approximately represents, for 3 of 5 conditions, half of the incentive for avoiding 1 readmission) and 1-SD changes in each of the incentives (by condition) and for the penalty (the hospital's reimbursement adjustment factor, ranging from no penalty to 3% penalty). A 2-sided *P* < .05 was set as the threshold for statistical significance.

There have been concerns that improvements in national readmission performance are attributable to regression to the mean. As a sensitivity analysis, we adjusted performance changes using a regression-to-the-mean adjustment^19^ using the methods of Barnett et al.^19^ This approach quantifies the effect of regression to the mean using variance components and the probability density and cumulative distribution functions of the standard normal distribution. These effects were computed for each of the 5 applicable conditions for hospitals with incentives (ERR >1) and without incentives (ERR ≤1). For hospitals with incentives, we additionally computed the regression to the mean according to the incentive tertiles.

## Results

We identified 2823 hospitals overall that participated in the HRRP during the baseline through follow-up years of the study. Of the 2823 hospitals analyzed, 2617 hospitals had ERR data for pneumonia, 1673 for AMI, 1284 for hip and/or knee surgery, 2576 for COPD, and 2651 for heart failure. A total of 2280 hospitals had ERRs greater than 1 for at least 1 applicable condition and 543 hospitals with ERRs 1 or less for all conditions in 2016.

### Readmission Avoidance Incentives Across Applicable Conditions

For hospitals with baseline ERRs that were greater than 1, readmission avoidance incentives varied considerably across conditions, including mean (SD) amounts of $8762 ($3699) for COPD, $9554 ($3322) for heart failure, $10 456 ($4642) for pneumonia, $16 874 ($7531) for AMI, and $58 158 ($26 198) for hip and/or knee surgery. Incentives also varied considerably by tertile. Mean (SD) incentives were approximately 3 times larger for tertile 3 vs tertile 1 for each of the conditions: $14 360 ($1440) vs $4938 ($3743) for pneumonia, $23 625 ($1899) vs $7736 ($5452) for AMI, and $81 532 ($7441) vs $26 690 ($19 594) for hip and/or knee surgery (Table 1). The differences in incentives between tertiles 2 and 3 were smaller. For example, mean (SD) incentives in tertile 2 were $12 072 ($530) for pneumonia and $19 285 ($1346) for AMI, $10 171 ($355) for COPD, $10 450 ($304) for heart failure, and $66 253 ($4073) for hip and/or knee surgery (approximately 15%-20% difference vs tertile 3 for each condition).

**Table 1.** Marginal Returns for Readmission Avoidance by Incentive Tertile and by Applicable Condition^a^

Condition	Tertile, mean (SD), $		
	First	Second	Third
Pneumonia	4938 (3743)	12 072 (530)	14 360 (1440)
Acute myocardial infarction	7736 (5452)	19 285 (1346)	23 625 (1899)
Hip and/or knee surgery	26 690 (19 594)	66 253 (4073)	81 532 (7441)
Chronic obstructive pulmonary disease	4391 (3193)	10 171 (355)	11 736 (870)
Heart failure	6085 (3524)	10 450 (304)	12 136 (1030)

^a^ The readmission avoidance financial incentive is the condition-specific financial outcome of an avoided readmission, reflecting the Hospital Readmissions Reduction Program penalty avoided and foregone revenue per each avoided readmission. Incentives were calculated from fiscal year 2016 Medicare data.

As shown in eFigure 1 in the Supplement, readmission avoidance incentives were also heterogeneous within each applicable condition. As shown in eFigure 2 in the Supplement, the incentives were moderately correlated with overall penalty amounts.

### ERRs and Readmission Avoidance Incentives

**Figure 1.**
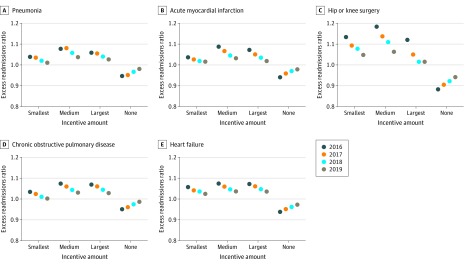
Readmission Performance From 2016 to 2019 by Hospitals’ Condition-Specific Incentive Amounts in 2016 per the Hospital Readmissions Reduction Program Excess readmissions ratio is a hospital’s condition-specific ratio of predicted-to-expected readmissions (ie, readmissions in excess of those expected given the hospital’s specific patient mix and the mean national hospital readmission performance level). The incentive tertile indicates the relative level of a hospital’s condition-specific marginal financial outcome of avoiding a readmission at baseline in 2016; this outcome consists of Hospital Readmissions Reduction Program penalty amount avoided as well as foregone patient revenue because of an avoided readmission. The penalty amount avoided for each condition was derived from the Hospital Readmissions Reduction Program statutory formula using differential calculus methods.

At the 2016 baseline, hospitals with greater incentives had larger ERRs. For pneumonia, ERRs were 1.08 for hospitals in tertile 2 and 1.06 in tertile 3 compared with 1.04 for those in tertile 1 and 0.95 for those with no incentives. For AMI, ERRs were 1.09 for hospitals in tertile 1 and 1.07 for hospitals in tertile 2 compared with 1.04 for hospitals in tertile 1 and 0.94 for the hospitals with no incentives. Observed associations of excess readmissions with incentive tertiles were similar for hip and/or knee surgeries, COPD, and heart failure conditions. Decreases in ERR during the next 3 years were observed for all targeted conditions, with ERRs narrowing by 2019 for higher- vs the lowest-incentive hospitals (Figure 1). For instance, in 2019, the AMI ERR was 0.3 percentage points (95% CI, –0.7 to 1.3 percentage points; *P* = .56 for the ERR difference between tertiles 3 and 1) to 1.6 percentage points (95% CI, 0.6 to 2.5 percentage points; *P* < .001 for the ERR difference between tertiles 2 and 1) higher for tertiles 2 and 3 compared with tertile 1, and 4.0 percentage points (95% CI, 3.1 to 4.9 percentage points) to 5.3 percentage points (95% CI, 4.4 to 6.1 percentage points) higher compared with no-incentive hospitals (*P* < .001); similar between-incentive category differences in ERR were observed for the other 4 targeted conditions.

### Change in ERRs by Levels of Readmission Avoidance Incentive

Excess readmissions were similar from 2016 to 2019 overall for 4 of 5 conditions. For hip fracture, there was a 2.9–percentage point overall decrease during this period (*P* < .001). The ERRs decreased for hospitals with incentives. For hip fracture, there was a 10.4–percentage point decrease (*P* < .001) for AMI and the ERR decreased 4.3 percentage points (*P* < .001); for COPD, the ERR decreased 3.8 percentage points (*P* < .001).

**Figure 2.**
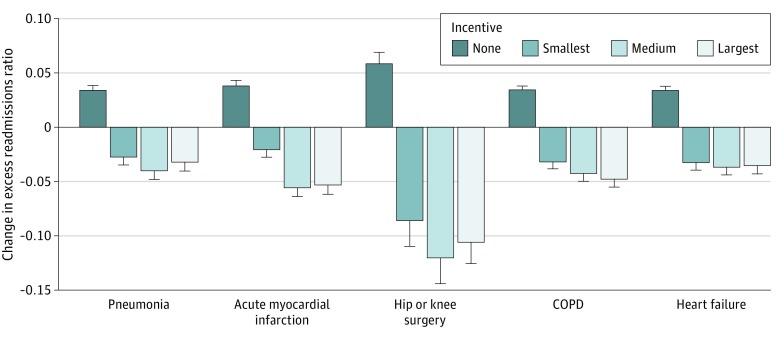
Change in Excess Readmissions Ratios by Incentive Size per the Hospital Readmissions Reduction Program From 2016 to 2019 Excess readmissions ratio is a hospital’s condition-specific ratio of predicted to expected readmissions (ie, readmissions in excess of those expected given the hospital’s specific patient mix and the mean national hospital readmission performance level). The incentive tertile indicates the relative level of a hospital’s condition-specific marginal financial outcome of avoiding a readmission at baseline in 2016; this outcome consists of Hospital Readmissions Reduction Program penalty amount avoided as well as foregone patient revenue because of an avoided readmission. The penalty amount avoided for each condition was derived from the Hospital Readmissions Reduction Program statutory formula using differential calculus methods. The no-incentive tertiles are tertiles of excess admissions among high-performing hospitals with no excess readmissions (excess readmissions ratio <1, no incentive for readmission avoidance) at baseline in 2016. COPD indicates chronic obstructive pulmonary disease; error bars indicate upper 95% confidence limits.

The decreases were larger for hospitals in the second- and third-incentive tertiles compared with the first tertile for all conditions (Figure 2). Across incentive tertiles, decreases in ERR from 2016 to 2019 were generally smaller for pneumonia (–2.8 percentage points in tertile 1 to –4.0 percentage points in tertile 2), COPD (–3.2 percentage points in tertile 1 to –4.2 percentage points in tertile 2), and heart failure (–3.3 percentage points in tertile 1 to –3.5 percentage points in tertile 3) and larger for AMI (–2.1 percentage points in tertile 1 to –5.0 percentage points in tertile 3) and hip and/or knee surgery (–8.6 percentage points in tertile 1 to –12.0 percentage points in tertile 2). Compared with higher-incentive hospitals in tertile 2, the lowest-incentive hospitals had smaller ERR decreases from 2016 to 2019 (45% smaller for pneumonia, 172% smaller for acute myocardial infarction, 40% smaller for hip and/or knee surgery, 32% smaller for COPD, and 13% smaller for heart failure).

Comparatively, for hospitals that had no incentives at baseline, performance worsened over time. The ERRs increased by 3.4 percentage points for pneumonia, 3.4 percentage points for COPD, 3.3 percentage points for heart failure, 3.8 percentage points for AMI, and 5.8 percentage points for hip and/or knee surgery (*P* < .001 for all). Given a baseline range of ERRs of 0.88 to 0.95, these results translated to increases in excess readmissions of 4% to 7% (median, 4% [percentage change of 3.7% for pneumonia, 4.2% for acute myocardial infarction, 7.1% for hip and/or knee surgery, 3.7% for COPD, and 3.7% for heart failure]; *P* < .001).

### Association of ERR Change With Incentive and Penalty Amounts

For hospitals with positive incentives at baseline, an additional $5000 increment in the readmission avoidance incentive was associated with statistically significant decreases in the ERR for 4 of 5 targeted conditions. The magnitudes ranged from 0.6 to 1.3 percentage points (Table 2), with the largest magnitudes of association for COPD (β [SE], –0.013 [0.002]; *P* < .001) and heart failure (β [SE], –0.013 [0.003]; *P* < .001). Given 6% (ERR, 1.06) and 7% (ERR, 1.07) excess readmissions at baseline for incentivized hospitals for these conditions, these translate to decreases of 22% (0.013/.06) for COPD and 26% (0.013/.05) for heart failure in excess readmissions for additional $5000 incentive increments.

**Table 2.** Amount of Change in Excess Readmissions Ratio From 2016 to 2019 Associated With Increments ($5000 and 1 SD) of the Baseline (2016) Marginal Return on Readmission Avoidance and the Baseline Penalty Amount^a^

Condition	Pneumonia (n = 1367)		Acute myocardial infarction (n = 914)		Hip and/or knee surgery (n = 696)		COPD (n = 1298)		Heart failure (n = 1345)	
	β (SE)	*P* value	β (SE)	*P* value	β (SE)	*P* value	β (SE)	*P* value	β (SE)	*P* value
Incentive of $5000	−0.007 (0.002)	.005	−0.012 (0.001)	<.001	−0.006 (0.001)	<.001	−0.013 (0.002)	<.001	−0.013 (0.003)	<.001
Incentive change, 1 SD	−0.006 (0.002)	.005	−0.018 (0.002)	<.001	−0.029 (0.006)	<.001	−0.010 (0.002)	.001	−0.009 (0.023)	<.001
Penalty change, 1 SD	−0.003 (0.002)	.22	−0.003 (0.002)	.12	−0.036 (0.006)	<.001	−0.008 (0.002)	<.001	−0.005 (0.002)	.009

Abbreviation: COPD, chronic obstructive pulmonary disease.

^a^ The readmission avoidance financial incentive is the condition-specific financial outcome of an avoided readmission, reflecting the Hospital Readmissions Reduction Program penalty avoided and foregone revenue per each avoided readmission. A β coefficient of –0.007 indicates that a $5000 increase in the readmission avoidance financial incentive was associated with a 0.7–percentage point decrease in the excess readmissions ratio for hospitals assessed as having any excess readmissions at baseline in fiscal year 2016. The penalty is proxied by the hospital's baseline error readmissions ratio.

As shown in Table 2, a 1-SD change in the incentive compared with the penalty was associated with substantially larger changes in ERR performance for pneumonia (incentive β [SE]: –0.006 [0.002], *P* = .005; penalty β [SE]: –0.003 [0.002], *P* = .22) and for AMI (incentive β [SE]: –0.018 [0.002], *P* < .001; penalty β [SE]: –0.003 [0.002], *P* = .12) and with slightly larger (25%) performance gains for COPD (incentive β [SE]: –0.010 [0.002], *P* < .001; penalty β [SE]: –0.008 [0.002], *P* < .001). However, a 1-SD change in the incentive compared with the penalty was associated with a 24% smaller performance gain for hip and/or knee surgery (incentive β [SE]: –0.029 [0.006], *P* < .001; penalty β [SE]: –0.036 [0.006], *P* < .001).

### Sensitivity Analysis

In a sensitivity analysis, we found that some of the observed change in performance from 2016 to 2019 could be attributed to regression to the mean; the findings were robust to this adjustment (Table 3). For instance, regression to the mean could explain 1.4–percentage point decreases for COPD and 3.6–percentage point decreases for hip and/or knee surgery from the baseline ERR for incentivized hospitals. Compared with the 3.8–percentage point (COPD) and 10.4–percentage point (hip and/or knee surgery) observed decreases, these represent 48% for pneumonia, 35% for AMI and hip and/or knee surgery, and 37% each for COPD and heart failure of observed decreases attributable to regression to the mean.

**Table 3.** Regression to the Mean in ERR Changes From ERRs in 2016^a^

Condition	Change in ERR, mean (SD), percentage points				
	Overall	First tertile	Second tertile	Third tertile	No incentive
Pneumonia	0.016 (0.012)	0.015 (0.011)	0.018 (0.013)	0.016 (0.012)	0.015 (0.013)
Acute myocardial infarction	0.015 (0.012)	0.011 (0.009)	0.017 (0.012)	0.018 (0.012)	0.015 (0.012)
Hip and/or knee surgery	0.036 (0.027)	0.034 (0.028)	0.039 (0.029)	0.033 (0.023)	0.026 (0.023)
COPD	0.014 (0.011)	0.012 (0.010)	0.016 (0.012)	0.015 (0.012)	0.012 (0.011)
Heart failure	0.013 (0.011)	0.012 (0.010)	0.014 (0.011)	0.014 (0.012)	0.013 (0.011)

Abbreviations: COPD, chronic obstructive pulmonary disease; ERR, excess readmissions ratio.

^a^ Values represent the mean percentage point change in the ERR that would be expected given 2016 ERR values because of the regression to the mean phenomenon. Tertiles 1 to 3 had an ERR greater than 1; no incentive had an ERR of 1 or less.

## Discussion

In this study, HRRP was associated with overall reductions in excess readmissions from 2016 to 2019 among hospitals with an initial penalty in 2016. This finding was consistent across conditions and procedures targeted by HRRP, with the largest reductions for hip and/or knee surgery and AMI. Decreases in readmissions were observed for hospitals in each of the incentive tertiles but were greatest for hospitals with larger initial incentive levels, with each additional $5000 in incentive associated with up to an approximately 25% decrease in ERR over time (approximately one-third of which was attributable to regression to the mean). Hospitals with no incentives at baseline had increased excess readmissions (4%-7%), meaning poorer performance during the 3-year period, across all of the targeted conditions. Results were robust to adjustment for regression to the mean and did not reflect administrative changes in diagnosis reporting introduced in 2011. Overall, hospitals with the largest incentives appeared to be most responsive to the program, which was consistent with the objective of policy efforts aiming to influence hospital behavior; even small incentive amounts were associated with observed decreases of more than 5% in excess readmissions.

Norton et al^10^ previously showed that improvement in performance was greatest among hospitals that had the largest incentives under Medicare's Value-Based Purchasing Program. Our similar finding that hospital performance was generally concordant with incentive size was novel in the readmission literature; however, because previous HRRP research has focused on penalty size and distribution^4,5,20^ and studies that have examined hospital responses to HRRP have focused on either overall performance^16^ or responses from specific hospital types (eg, safety-net, teaching, and minority group–serving hospitals).^5,14,15^ We found that hospitals were responsive to HRRP's penalty but appeared to be most responsive to future condition-specific marginal returns, which differed from the penalty in that they included potential foregone revenue from avoided readmissions and because the incentive accounted for caps on the size of the penalty for the worst-performing hospitals. This finding was consistent with hospitals having some awareness of the incentives and the components (eg, foregone revenue, caps) that they contain.

Greater improvements in performance for several of the HRRP-targeted conditions, particularly hip and/or knee surgery, were consistent with documentation in an earlier report.^21^ They were also consistent with the size of the HRRP incentive, which was more than 5 times larger for hip and/or knee surgery than for other conditions.^12^ Large reductions were also observed for AMI, which similarly had larger incentives compared with the other targeted conditions. The observed decreases in ERRs among incentivized hospitals for hip and/or knee surgery and COPD were also noteworthy given that those 2 conditions were introduced into the HRRP in 2015, meaning hospitals may have had less time to implement condition-specific protocols for addressing patients discharged with those conditions.

Earlier research showing reductions in national readmissions after HRRP implementation^13,21^ has been called into question by evidence that nonperformance factors are associated with inflated observed reductions in risk-standardized readmission ratios over time.^22,23,24^ These include administrative changes in Medicare's processing of secondary diagnosis codes. Ody et al^22^ reported that risk-adjusted readmission rates appeared to diminish over time because of more secondary diagnoses collected by Medicare beginning in January 2011. However, our study period (beginning October 2011) did not overlap with that Medicare policy change; thus, patient severity changes reflecting this Medicare administrative policy change should not have affected our findings.

Other researchers have reported that upcoding is responsible for a large proportion of observed improvement in performance under HRRP,^24^ but in our study, such bias might be toward the null given that hospitals with smaller baseline incentives may be more likely to upcode (with the assumption that better-resourced hospitals perform better and have the administrative capacity to code claims most efficiently).

There was no evidence that hospitals would choose to sustain the penalty rather than forego reimbursement from the readmission^25^; if this were the case, we would not have observed such consistent responsiveness to the incentives. Discontinuities in hospital responses at the upper incentive levels occurred, with slightly improved performance for tertile 2 compared with tertile 3 hospitals. The differences in incentives for hospitals in these 2 tertiles were small, which may have limited response variation. Alternatively, the results may support the argument that the highest-incentive hospitals were limited in their ability to effectuate prevention.

For policy makers, the results may indicate the need for recalibration of the incentives. The ability of HRRP to optimize hospital performance may be muted because of the program's lack of a carrot. This penalty-only structure mutes incentives as performance improves. By not providing a positive inducement for change, for better-performing hospitals, HRRP may be restricting potential gains in overall performance. The yardstick for HRRP was mean national performance; therefore, if the best-performing hospitals worsen, the expected performance in future years for other hospitals would also lower. Over time, this could harm quality efforts in better-performing hospitals and other hospitals because of lowered incentives.

### Future Directions

The findings suggest that incremental incentives are associated with performance improvement for the HRRP. To the extent that other factors may coincide with observed performance improvements according to incentive amounts, future research should examine the stability of patient populations among hospitals with differing incentive levels. It may also be important to examine whether hospitals with the largest incentives may have had changes in their underlying patient populations given concerns that HRRP may encourage underresourced hospitals to stop treating at-risk patients who are more likely to be readmitted. Addressing these concerns may optimize overall readmission performance in US hospitals.

### Limitations

This study has limitations. First, results partly reflect regression to the mean, in which the values of repeated ERR measurements decreased or increased according to whether they were initially above or below the mean.^26,27^ However, although substantively diminished in magnitude, our results were nonetheless robust to adjustments for this statistical phenomenon. Second, we were unable to compute hospitals’ specific penalties when assessing the association between the penalty amount and hospital performance change; to do so would require additional (claims) data. However, the adjustment factor that we used in place of the penalty (ie, no adjustment to a capped 3% adjustment) is a strong proxy for the penalty (given that the penalty is the adjustment factor standardized by the ratio of spending on readmissions to all discharges). Hospitals may have used observation unit stays to avoid index hospitals that could result in readmissions to avoid penalties, but previous work suggests that such approaches were not associated with decreases in national readmissions after implementation of HRRP.^13^

## Conclusions

In this study, the penalty incentives in HRRP were associated with improvements in readmission avoidance. However, hospitals performing better initially had more muted gains, suggesting a potential need for strengthening their incentives under HRRP.

## Notes

Supplement. **eAppendix.** Reasons for Heterogeneity of Marginal Financial Impacts Under HRRP **eFigure 1.** Distribution of Marginal Returns From an Avoided Readmission, by Applicable Condition **eFigure 2.** Correlations Between Condition-Specific Incentives and Overall Penalty Amounts
