# Supplementary material for: Association Between Financial Incentives in Medicare's Hospital Readmissions Reduction Program and Hospital Readmission Performance
Source: JAMA Netw Open. 2020 Apr 3;3(4):e202044. doi: 10.1001/jamanetworkopen.2020.2044 (PMC7125432; doi:10.1001/jamanetworkopen.2020.2044)
Supplement: Supplement. — eAppendix. Reasons for Heterogeneity of Marginal Financial Impacts Under HRRP eFigure 1. Distribution of Marginal Returns From an Avoided Readmission, by Applicable Condition eFigure 2. Correlations Between Condition-Specific Incentives and Overall Penalty Amounts [file jamanetwopen-3-e202044-s001.pdf]

Supplementary Online Content

Hoffman GJ, Yakusheva O. Association between financial incentives in Medicare's Hospital Readmissions Reduction Program and hospital readmission performance. *JAMA Netw Open*. 2020;3(4):e202044. doi:10.1001/jamanetworkopen.2020.2044

eAppendix. Reasons for Heterogeneity of Marginal Financial Impacts Under HRRP

eFigure 1. Distribution of Marginal Returns From an Avoided Readmission, by Applicable Condition

eFigure 2. Correlations Between Condition-Specific Incentives and Overall Penalty Amounts

This supplementary material has been provided by the authors to give readers additional information about their work.

© 2020 Hoffman GJ et al. *JAMA Network Open.*

eAppendix. Reasons for Heterogeneity of Marginal Financial Impacts Under HRRP

Heterogeneity in these marginal financial impacts are due to characteristics of the penalty and the provider. First, the penalty's cap and floor effects create discontinuities in incentives not seen in ERRs or the penalty. The incentive can also vary according to hospital volumes, patient mix, and Medicare reimbursement levels. Because, the penalty is a ratio of spending on excess readmissions and total spending for all discharges, all else equal hospitals with higher volume see smaller penalty reductions for an avoided readmission. Also, hospitals with higher labor costs or that treat low-income patients potentially forego more patient revenue for an avoided readmission. This is because Medicare reimburses more for hospitals with higher labor costs through its wage index and for hospitals treating more low-income patients or employing medical trainees, each of which is not penalized under HRRP; therefore, hospitals retain these supplemental reimbursements even if they readmit a patient.

eFigure 1. Distribution of Marginal Returns from an Avoided Readmission, by Applicable Condition

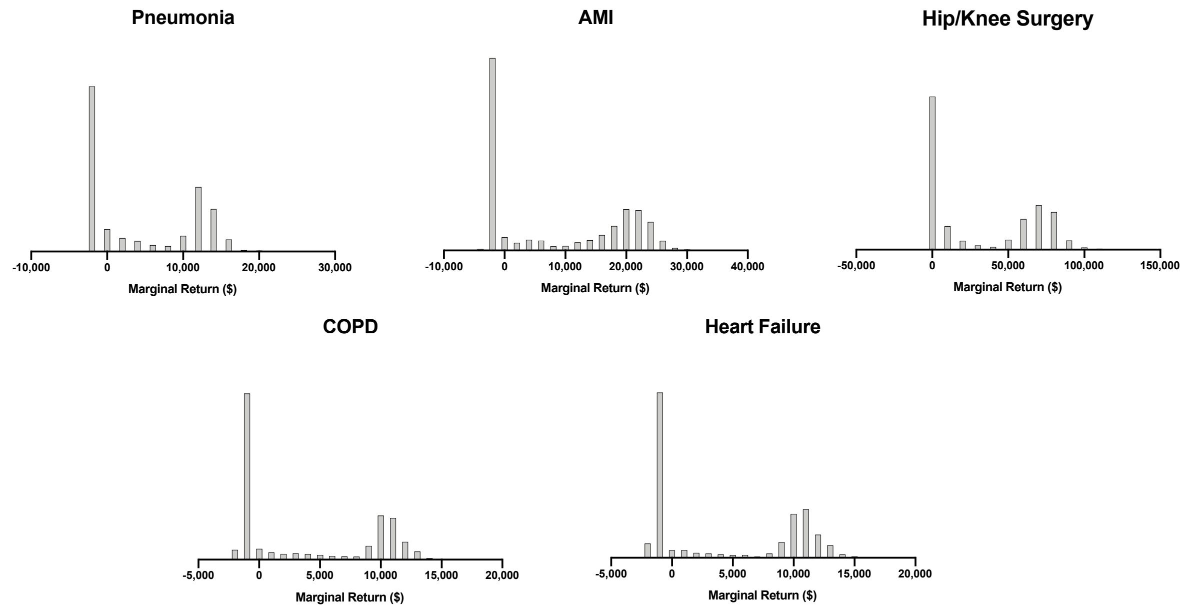

Notes: AMI = acute myocardial infarction; COPD = chronic obstructive pulmonary disease. The marginal return from an avoided readmission for each condition was derived from the HRRP statutory formula using differential calculus methods and by assessing the patient revenue foregone from each avoided readmission.

eFigure 2. Distribution of Marginal Returns from an Avoided Readmission, by Applicable Condition

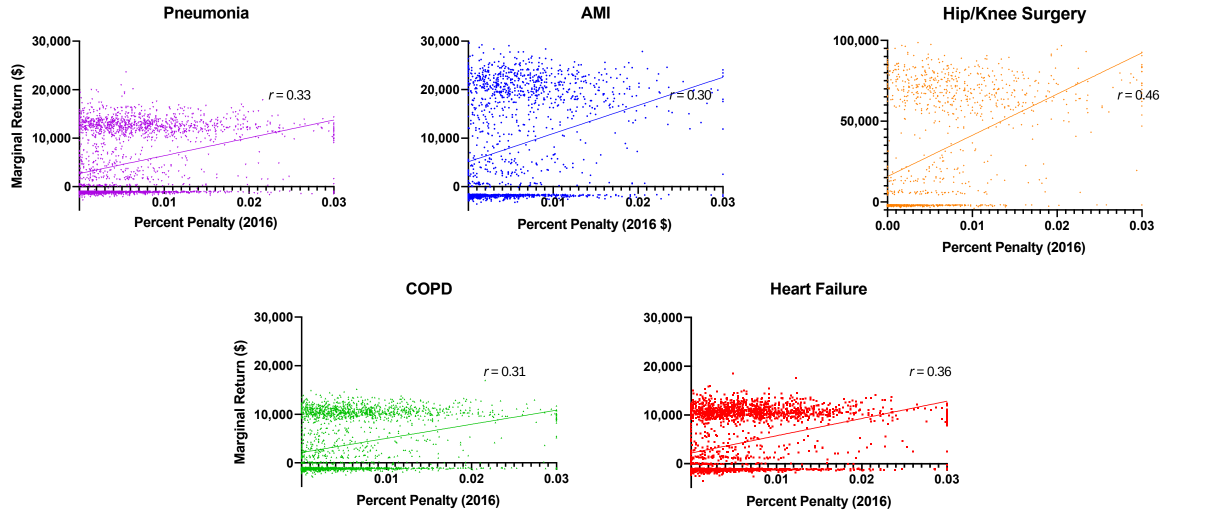

Notes: AMI = acute myocardial infarction; COPD = chronic obstructive pulmonary disease. The marginal return from an avoided readmission for each condition was derived from the HRRP statutory formula using differential calculus methods and by assessing the patient revenue foregone from each avoided readmission. Linear trend lines are shown for each condition, representing the relationship between the 2016 HRRP penalty and the computed marginal returns for an avoided readmission. The Pearson correlation coefficients (*r*) are also shown for each condition.

© 2020 Hoffman GJ et al. *JAMA Network Open.*
